# Supplementary material for: Metabolic Syndrome Prevalence in Women with Gestational Diabetes Mellitus in the Second Trimester of Gravidity
Source: J Clin Med. 2024 Feb 22;13(5):1260. doi: 10.3390/jcm13051260 (PMC10932344; doi:10.3390/jcm13051260)
Supplement: Supplementary file 1 [file jcm-13-01260-s001.zip › jcm-2863121-supplementary.pdf]

Supplementary Table S1—The list of the clinical centres participated in this study.

**Clinical Center**	**Number of Women**
Diabetes Centre, Internal clinic of the Faculty Hospital Brno (Jihlavska 20, Brno, Czech Republic),<br><br>web: https://www.fnbrno.cz/en/department-of-gastroenterology-and-internal-medicine/k1450	Enrollment of 455 GDM subjects (all participants of the study)
Obstetric and Gynaecology clinic of the Faculty Hospital Brno (Jihlavska 20, Brno, Czech Republic<br><br>Web: https://www.fnbrno.cz/en/maternity-hospital/t6211	Peripartal data 295 (65% of participants of the study)
